# Critical Understanding of the Influence of Cellular Aging Biomarkers on Host–Parasite Relationships Serving as a Key Platform for Malaria Eradication

**DOI:** 10.3390/biology14101458

**Published:** 2025-10-21

**Authors:** Dorathy Olo Anzaku, Israel Sunmola Afolabi

**Affiliations:** 1 College of Science and Technology, Covenant University, Ota 112104, Ogun State, Nigeria; israel.afolabi@covenantuniversity.edu.ng; 2 Covenant Applied Informatics and Communication Africa Center of Excellence, Covenant University, 4th Floor, Right-Wing, CUCRID Building, Ota 112104, Ogun State, Nigeria

**Keywords:** host–parasite relationship, parasite, malaria, plasmodium parasite, aging biomarkers

## Abstract

Plasmodium parasites are the causative agents of malaria and can infect humans and other vertebrates, impacting socioeconomic development and causing significant health issues globally. *Plasmodium falciparum* causes the most severe type of infection, which can lead to chronic morbidity and other severe complications like anemia and cerebral malaria. The onset of infection is marked by the injection of sporozoites into the skin through the bite of a female Anopheles mosquito. This triggers a cascade of reactions elicited both by the host immune system in response to infection and by the parasite in a bid to evade the host immune system, survive, and replicate. The dynamics of this host–parasite relationship have prompted extensive research in an attempt to understand and exploit it in the fight against malaria. Thus, understanding the temporal and spatial dimensions of adaptation in host–parasite relationships is critical for forecasting parasite evolution and spread within and between host populations. One such relationship is the complex interplay between malaria and cellular aging processes. Understanding this dynamic will provide novel insights into the pathophysiology of the disease. This comprehensive review takes us on that journey by providing an overview of the interaction between the Plasmodium parasite and its host and the interplay between infection mechanisms, host immune response, and parasite evasion strategies, narrowing it down to how it affects cellular aging biomarkers and how this can be explored as a platform in the fight against the disease.

## Simple Summary

Malaria is a disease caused by Plasmodium parasites, and it spreads to humans through the bite of infected female Anopheles mosquitoes. *Plasmodium falciparum* is the deadliest of the five species responsible for transmission. In the human host, the parasite goes through various stages in an attempt to multiply and cause infection. While the host’s immune system mounts a response to fight the infection, the parasite employs various evasion mechanisms to escape and conquer the host. This host–parasite relationship is considered complex because it is influenced by numerous factors, making some aspects difficult to understand. One such aspect is how malaria infection affects cellular aging in its human host. Oxidative stress and inflammation are reportedly key drivers of this process, occurring in response to infection and leading to the production of reactive oxygen species (ROS). This process damages cells, contributing to the shortening of telomeres, which are the protective caps on chromosomes that regulate the cell’s lifespan. Subsequently, this can lead to premature aging and a weakened immune system. The caveat, however, is that understanding these processes is crucial for developing strategies to mitigate the long-term effects of malaria.

## 1. Introduction

Malaria, a disease caused by the plasmodium parasite, spreads to humans through the bites of female anopheline mosquitoes. Though preventable and curable, 300–500 million new cases and 1.5–2.7 million fatalities are recorded annually, which makes malaria a serious worldwide health concern [1]. Human malaria is caused by mainly five parasitic species, two of which, *Plasmodium falciparum* (*P. falciparum*) and *Plasmodium vivax* (*P. vivax*), are the most dangerous. Malaria is primarily found in tropical and subtropical regions of the world, and those most vulnerable to the illness are pregnant women and children under five years of age [2], who account for the highest mortality. Fever, headache, and chills are usually the first symptoms, evident 10–15 days after the infective mosquito bite. On the African continent, *P. falciparum* causes the deadliest and most common form of malaria, whereas in most places outside of Sub-Saharan Africa, *P. vivax* is the most common malaria-causing parasite [3]. Meanwhile, although vector control methods like insecticide-treated nets (ITNs) and indoor residual spraying (IRS) have proven successful in lowering malaria transmission rates [4], they are quickly losing effectiveness due to growing vector resistance, which is attributed to P450 enzymes that have been linked to the detoxification of various insecticides, especially pyrethroid resistance [5]. P450 enzymes also overproduce detoxifying enzymes such as glutathione S-transferase, carboxy cholinesterase, and monooxygenase P450, which leads to metabolic resistance. As a result of these events, numerous pyrethroid-resistant malaria vectors in Africa have been found to exhibit high levels of P450 activity, thus decreasing the efficiency of malaria control measures by vectors that include enzymes that increase resistance to pyrethroids and carbamates, such as CYP6M2 and CYP6P3 in *Anopheles gambiae* and CYP6P9a and CYP6P9b in *Anopheles funestus* [6,7]. Additionally, the P450 enzyme has wide substrate activity, which has raised concerns about cross-resistance to other pesticides, which could jeopardize malaria control efforts. Successful malaria control requires tactics like resistance allele tracking, piperonyl butoxide (PBO)-based nets, and insecticide rotation. Additionally, drug resistance is a significant issue in the treatment of malaria, with resistance to anti-malarial medications such as chloroquine, pyrimethamine, mefloquine, and sulfadoxine-pyrimethamine reported globally [8,9]. For uncomplicated *P. falciparum* malaria, artemisinin-based combination therapies (ACTs) are utilized as first and second-line treatments [10]. However, it has been discovered that the Greater Mekong subregion and some African countries, including Rwanda and Uganda, exhibit resistance to artemisinin. While resistance to artemisinin alone seldom results in treatment failure, resistance to both artemisinin and companion medicine in ACT therapy regimens can cause significant treatment failure rates [3]. Although reported to be partial, ART resistance has emerged in Africa, and this has been linked to a mutation in the K13 gene [11]. As a result of this, there appears to be reduced parasite clearance, which means potential treatment failure could arise. While contradictory findings about the efficacy of ACTs require more research [10], studies are also underway to find potential drug targets [12,13]. Furthermore, chemoprevention strategies, such as intermittent preventive treatment during pregnancy (IPTp), in infants (IPTi), and seasonal malaria chemoprevention (SMC), have been shown to reduce malaria-related illness and death [8]. However, the effectiveness of anti-malarial drugs used in these strategies is increasingly challenged by the development of drug resistance. As a result, recommendations for chemoprevention may need to shift toward broader guidelines rather than specific resistance marker thresholds, particularly when considering whether current or future treatment drugs share resistance mechanisms with those used in chemoprevention. The development of vaccines is an essential area of research, and clinical tests have shown that the RTS,S/AS01 malaria vaccine produces positive results. This has been approved by the World Health Organization and is actively employed for use in children. Another approved vaccine is the R21/Matrix-M. Both have reportedly been efficacious in preventing malaria, as uncomplicated malaria is said to have reduced by 40%. A 30% reduction was also reported for severe malaria and 13% for mortality according to the Centre for Disease Control (CDC). Test programs for these vaccines have been conducted in various African countries with reports of successful reduction in malaria incidence in endemic areas [14,15]. The development of drug and pesticide resistance poses a serious challenge to the treatment and prevention of malaria. Drug resistance makes it necessary to develop new strategies to combat anti-malarial resistance, as it threatens the efficacy of conventional malaria treatment alternatives like the use of ACTs and chemoprevention approaches. The effectiveness of vector control techniques, such as insecticide-treated nets (ITNs) and indoor residual spraying (IRS), in stopping the spread of malaria is significantly hampered by pesticide resistance. Both of these necessitate further research to develop immunizations that can provide long-term protection against malaria, as research on vaccine development is crucial.

## 2. An Overview of Immunity in Malaria

Human malaria is caused by Plasmodium parasites that are deposited into the skin after a blood meal by an infected female Anopheles mosquito [1]. The immune response is complicated because of the parasite’s unique life cycle in the human host. In the human host, the parasite travels through two primary stages: the liver stage and the blood stage. Each stage causes a different immunological response. There is substantial documentation of the parasite’s life cycle in humans, including its comprehensive progression [1,16]. The immune response to malaria infection can be categorized into two types: natural immunity and acquired immunity.

Plasmodium parasite exposure over time leads to the development of natural immunity to malaria. Although it offers some defense, this type of immunity is not enough to curb infection [17]. Adaptive and innate immune responses are both components of natural immunity. The innate immune response, which includes the activation of natural killer (NK) cells, macrophages, and the generation of pro-inflammatory cytokines like interferon-gamma (IFN-γ), is the first line of defense against Plasmodium parasites [18]. Additionally, the innate immune system plays a crucial role in activating the adaptive immune response. Due to their underdeveloped immune systems, children in malaria-endemic areas typically develop immunity more slowly, leaving them more susceptible to severe malaria [17]. It has been demonstrated that genetic variables, such as hemoglobinopathies and the Dantu red blood cell variation, confer resistance to malaria, lowering infection rates and disease severity [18].

Hemozoin (HZ) is one of the parasite-derived immune-stimulatory ligands that have been discovered through studies of the innate immune response during the blood stage of malaria. The crystalline-insoluble result of hemoglobin breakdown, HZ, has immunomodulatory qualities [19] and may have both pro- and anti-inflammatory effects, according to previous studies [20], and its buildup in tissues has been connected to the severity of malaria in both patients and animal models. Notably, the presence of HZ-carrying monocytes and granulocytes has been positively connected with malaria-associated anemia. However, several studies reveal varying immunological responses, indicating that the effects of HZ are still context-dependent. HZ has been shown to affect immunological responses over time by inducing the production of cytokines and chemokines by dendritic cells and macrophages, which in turn modifies the host immune system. The exact mechanisms behind these impacts are still mostly unknown, though. The immunological effects of hemozoin, including how it contributes to the pathophysiology of cerebral malaria by causing oxidative stress and apoptosis in neurons and astrocytes, have been reviewed [21]. Additionally, during parasite development, higher HZ levels have been linked to an increase in 4-HNE conjugates, which are indicators of oxidative damage [22].

Adaptive immunity against malaria is mediated by T and B cells and involves antigen-specific responses. B cells and macrophages are essential for regulating the erythrocytic stage, and CD4+ T helper cells are essential for their activation [18,23]. The production of antibodies by B cells, such as anti-Plasmodium IgG, is crucial for eliminating parasites and averting serious illness [24].

The primary way that macrophages, dendritic cells (DCs), and natural killer (NK) cells contribute to the innate immune response is by releasing cytokines that promote inflammation [25]. The functions of these cells are outlined in Table 1. CD4+ T cells mediate cellular and antibody-driven immunity after they have been activated and develop into Th1 and Th2 subsets. Through processes including opsonization and phagocytosis, complement-mediated lysis, and antibody-dependent cell-mediated destruction, the generation of antibodies aids in the removal of merozoites and parasitized red blood cells (pRBCs).

Additionally, NK cells have been implicated in malaria immunity, especially in early-stage infections before CD4+ T cell activation; research in malaria-endemic areas indicates that NK cell activation takes place early in the infection process [26]. The adaptive immune response relies heavily on cytotoxic T cells and B cells. Antibodies promote complement-mediated lysis, which facilitates the removal of gametocytes from the mosquito vector. Furthermore, the removal of gametocytes has been linked to nitric oxide.

*P. falciparum* evades host immunological responses by secreting virulence proteins and attacking host erythrocytes, among other immune evasion strategies [27]. A secretome in a malaria infection is revealed by *P. falciparum* virulence and remodeling proteins, which target host erythrocytes [27]. In situ, switching, mutually exclusive var gene transcription, and the distinct location of the tiny variant STEVOR antigen within Maurer’s clefts in infected red blood cells are all examples of antigenic diversity in malaria. These methods entail suppressing macrophages and avoiding immune detection through interactions with inhibitory immune receptors, including LILRB1 and LILRB2. It is essential to comprehend immune evasion processes to create malaria vaccines that effectively target the parasite’s capacity to elude host immunological responses. The pathogeneses and immunity to *P. falciparum* malaria rely heavily on *Pf*EMP1, a critical variant surface antigen. The ability of virulence genes to be transcriptionally flexible allows malaria parasites to change and elude host defenses [28]. A study reported that the variety in regional pools influencing the host’s immune response is influenced by the rate of gene substitution in specific local populations [29].

**Table 1:** Immune response to malaria at different stages of infection, along with key immune cells involved and their functions.

Stage of Infection	Immune Cells	Function	References
Pree-Erythrocytic stage (Sporozoite invasion and liver stage)			
	Dendritic cells (DCs)	Recognize and process Plasmodium antigens. During the pre-erythrocytic stage, deliver antigens to T cells to trigger CD4+ and CD8+ CELL RESPONSES, hence triggering adaptive immunity.	[26,30]
	Natural Killer (NK)cells	pRBC destruction and the release of pro-inflammatory cytokines, such as IFN-γ, to stimulate macrophages	[31]
	Macrophages	Initiate an immunological response by phagocytosing infected hepatocytes.	[18]
	CD8+ cells	function during the infection’s liver stage. Identify parasite antigens on infected hepatocytes using MHC, then get rid of those cells.	[32]
Erythrocytic Stage			
	Neutrophils	Usually the first responders, they emit reactive oxygen species (ROS) and phagocytose-contaminated red blood cells (RBCs).	[33]
	Monocytes/macrophages	Infected hemozoin and RBCs are phagocytosed, and pro-inflammatory cytokines (TNF-α, IL-1β) are produced. generate NO and ROS, which are poisonous to parasites.	[34]
	CD4+ Helper T cells	Targeting circulating merozoites and pRBCs, TH2 CD4 cells stimulate B cells to generate antibodies, particularly immunoglobulin G, which sets off a series of events. Control the inflammatory reaction. both antibody-mediated and cell-mediated immunity. By assisting macrophages in eliminating parasitized red blood cells, Th1 AIDS macrophages support cell-mediated immunity. Produce cytokines such as interferon gamma, enhancing CD8+ function	[32]
	B cells and Plasma cells	They facilitate the production of antibodies against parasite antigens, such as MSP1 and *Pf*EMP1, and also facilitate the removal of parasites through opsonization and phagocytosis. Prevent the invasion of uninfected RBCs by merozoites	[35,36]
Cerebral Malaria			
	T cells (CD4+ and CD8+)	contribute to disease caused by the immune system by causing excessive inflammation.	[37]
	Brain macrophages	contribute to blood-brain barrier disruption and mediate neuroinflammation.	[38]
	Endothelial cells	In cerebral arteries, upregulation of adhesion molecules (ICAM-1, VCAM-1) results in the sequestration of parasites.	[39]
Chronic and Adaptive immunity			
	Memory T Cells (CD4+ and CD8+)	Respond to upcoming illnesses to establish long-term immunity. During the blood stage, CD8+ T cells are able to identify and eliminate infected red blood cells that have antigens on their surface. also increases macrophage activity and generates cytokines like interferon gamma.	[31]
	Memory B cells	aid in the quick development of antibodies after reinfection.	[17]
	Regulatory T cells	Control the immune response to avoid immunological fatigue and excessive inflammation.	[21]

## 3. Host–Parasite Relationship in Malaria

Life within the host intracellular milieu demands a significant level of commitment to defend against host defenses and guarantee a consistent supply of nutrients. The plasmodium parasite that causes malaria belongs to the Phylum Apicomplexa, which is made up of obligatory intracellular protozoan parasites that enter and disrupt their hosts’ cells to carve out a space for themselves to reproduce [40]. The host–parasite relationship in malaria sickness fluctuates due to many factors, such as co-infections, human polymorphisms, environmental determinants, and the genetic diversity of important parasite proteins. The plasmodium parasite, like most apicomplexan parasites, lives its internal life stages inside a parasitophorous vacuole (PV), created when the parasite invades the host cell’s plasma membrane. The PV offers a protective environment to establish and preserve the intracellular niche, but it also requires the coordination of many transport activities across this barrier [41,42].

The following factors contribute to variation in the host–parasite relationship in malaria;

(A) Parasite genetic diversity of key proteins: The host’s immunological response and the parasite’s capacity to elude it can be influenced by the genetic variety of important proteins in the parasite, such as the surface proteins. Variations in the parasite’s virulence and the host’s vulnerability to infection may result from this variety [43,44]. The ability of *Plasmodium falciparum* to elude the host’s immune system and adapt to its environment is facilitated by the genetic diversity of important proteins, including the glutamate-rich protein (GLURP) and merozoite surface proteins (MSPs) 1 and 2. Because it enables the parasite to adapt and alter its surface antigens to evade immune system detection, this diversity is essential to the parasite’s survival and spread.

A study examined the transport systems that function at the interface between the host and parasite during the blood stage of malaria, highlighting the wide range of effector proteins that blood-stage malaria parasites export to extensively modify the host cell [45]. This remodeling allows the parasite to establish a favorable intracellular environment [46]. Other studies have also characterized some of these proteins [47,48]. It is believed that in *Plasmodium falciparum*, the most dangerous malaria parasite in humans, there are over 500 of these effector proteins. Many of these proteins are members of multigene families, including sub-telomeric variable open reading frame proteins (STEVORs), repetitive interspersed family proteins (RIFINs), and *P. falciparum* erythrocyte membrane protein 1 (*Pf*EMP1). These proteins are embedded into the membrane of infected red blood cells (iRBCs), facilitating their attachment to the endothelial cells lining the capillaries, which helps sequester iRBCs from circulation and prevents them from being filtered by the spleen [27]. The spleen plays a significant role in the interaction between the host and the parasite. Research has shown that *P. vivax* levels increase following splenectomy, while *P. ovale* shows a consistent rise in the blood stage [49].

It has also been discovered that *P. falciparum* lacks the enzymes required for RNA interference and relies on enzymes imported from human red blood cells to regulate its gene expression [46]. MicroRNAs (miRNAs) are critical in modulating immune cell responses to infections. Although the exact roles of many miRNAs remain unclear, with some being upregulated and others downregulated in various studies, miR-223 and miR-19b were found to be upregulated in the red blood cells of malaria-infected patients [46]. miRNAs may serve as biomarkers for detecting malaria since the parasite influences the host’s miRNA expression. miRNAs are involved in many biological processes and are primarily found in the non-coding areas of the genome. They control post-transcriptional levels of gene expression. The function of miRNAs in malaria has been investigated in some studies utilising animal models. For instance, changes in miRNA expression were seen in mice infected with *P. chabaudi* and P. ANKA, which helped the animals build immunity against the parasite. Additionally, compared to non-cerebral malaria and uninfected controls, the brains of *P. berghei*-infected mice, which induced cerebral malaria, showed increased expression of miR-27a, miR-150, and let-7i [50]. This implies that pathways related to both innate and adaptive immunity may be regulated by miRNAs.

Further on in the diversification, the generation of reactive oxygen species (ROS) by x-ray irradiation results in single-strand and double-strand breaks in the DNA. Elevated ROS thus causes antigen diversification in *P. falciparum*, so linking the host immune response to the process of multicopy antigen gene family diversification in malaria parasites [51]. Targeting human erythrocytes, parasite virulence, and remodeling proteins exposes a secretome in malaria infection. In situ switching, mutually exclusive var gene transcription, and the distinct location of the tiny variant STEVOR antigen within Mayrer’s clefts in infected RBC are all examples of antigenic diversity in malaria [51]. *P. falciparum* utilizes immune evasion mechanisms, such as targeting host erythrocytes and secreting virulence proteins, to evade host immune responses. These strategies involve interactions with inhibitory immune receptors such as LILRB1 and LILRB2 to suppress macrophages and evade immune detection [36,52]. *Pf*EMP1 is a key variant surface antigen important in *P. falciparum* malaria immunity and pathogenesis. The transcriptional plasticity of virulence genes allows malaria parasites to adapt and evade host immunity. Zhan et al. (2024) reported that the substitution rate of genes in individual local populations contributes to the diversity in regional pools, affecting the host immune response [29]. *P. falciparum* exchanges metabolites with the host for proliferation, diverting nutrients from the host toward its growth. The parasite is found within mature erythrocytes in the blood and relies on the breakdown of hemoglobin and extracellular amino acids. The changes in red blood cell metabolism induced by the parasite occur during the developmental cycle, affecting nutrient utilization [53].

Evasion strategies involve the genetic diversity of key proteins in *P. falciparum*, allowing the parasite to continuously alter its surface antigens to elude the host’s immune response [54]. This diversity allows the parasite to produce novel combinations of alleles, which can benefit the parasite’s survival and transmission [43,44,55]. It also enables the parasite to adapt to the host environment. For example, the parasite can develop resistance to anti-malarial drugs by evolving genetic mutations that make it less susceptible to these treatments [8,56]. The genetic diversity of key proteins can also lead to polyclonal infections, where an individual is infected with multiple genetically distinct parasite variants [57,58]. This can result in a more severe disease outcome and increased transmission potential. This is driven by evolutionary selection, where beneficial genetic variations are favored and spread through the parasite population [59]. This process maintains the genetic diversity of the parasite and allows it to adapt to changing host environments.

The following are the impacts it has on the host–parasite relationship:

1. Host immune response: The genetic diversity of key proteins in *P. falciparum* influences the host’s immune response. The constant change in surface antigens hinders the host’s ability to mount a successful immune response, allowing the parasite to evade recognition and continue to infect the host.
2. Disease severity: It can also impact the severity of malaria. Polyclonal infections, for example, can lead to more severe disease outcomes due to the increased number of parasite variants engaging with the immune system of the host.
3. Transmission dynamics: It affects the transmission dynamics of malaria. The ability of the parasite to adapt to the host environment and evade the immune system increases its transmission potential, contributing to the persistence of malaria in human populations.

(B) Co-Infections: Co-infections with other pathogens or parasites can alter the host’s immune response and the parasite’s ability to establish and maintain infection. This can result in variations in the disease’s severity and the host’s ability to recover [1]. Co-infection in malaria refers to the simultaneous infection of an individual or host with multiple strains or species of malaria parasites, which can have significant implications for disease severity and treatment outcomes [60]. Co-infection can increase genetic diversity within the parasite population, potentially impacting the effectiveness of host immune responses and treatment strategies. Co-infection has been found to impact parasitemia, inflammation, anemia, and erythrocytes with parasites sequestered in the brain’s microvasculature, all of which can impact the severity of malaria [61].
(C) Delays in treatment: Delays in seeking medical attention or receiving effective treatment can allow the parasite to establish a stronger foothold in the host, leading to more severe disease outcomes. This delay can also increase the risk of developing resistance to anti-malarial drugs [62]. Delays in the treatment of uncomplicated malaria can significantly impact the host–parasite relationship and increase the risk of developing severe disease. Compared to those treated within 24 h, children and adults who experienced delays longer than 24 h from the onset of symptoms were significantly more likely to acquire any severe malaria phenotype [63]. Longer delays were linked to considerably greater dangers in SMA, such as delays of two to three days as opposed to therapy within twenty-four hours. Researchers predict that if everyone sought therapy on the first day of symptoms, 42.8% of child SMA cases and 48.5% of adult SMA cases may have been avoided [63].

Delays in the clearance of parasites after starting treatment are multifactorial, related to drug resistance, and linked to treatment failure. Independent risk factors include young age (≤2 years), high initial parasitemia (>50,000/μL), fever, and treatment with non-artemisinin monotherapies [64]. Gametocyte carriage rises dramatically when parasite clearance is delayed, which affects attempts to stop malaria from spreading.

(D) Host genetic variations, such as polymorphisms in genes related to immune response, can affect an individual’s vulnerability to malaria and the intensity of the disease [65]. These genetic differences may also influence how effectively the host responds to anti-malarial therapies [1]. Research indicates that genetic variations in the human host can impact susceptibility to malaria and the severity of the disease [65]. Studies suggest that certain genetic mutations and polymorphisms in humans provide a survival benefit against malaria, which has led to their increased frequency through natural selection. Notable examples of these variations include the sickle cell trait (HbAS), thalassemias, and glucose-6-phosphate dehydrogenase (G6PD) deficiency [66,67,68]. These genetic changes can modify the immune system’s response or disrupt host–parasite interactions, thereby contributing to differences in malaria manifestations and influencing the disease’s pathology. Additionally, recent advancements in genomic research, such as genome-wide association studies (GWAS), have pinpointed various genetic polymorphisms linked to either heightened susceptibility or resistance to malaria. While certain polymorphisms have been shown to play important roles in malaria susceptibility, the results are often inconsistent and may differ across different populations [61].
(E) Environmental factors such as climate, altitude, humidity, temperature, and proximity to water bodies can influence the distribution and prevalence of malaria. Factors like humidity, precipitation, temperature, and proximity to permanent water bodies significantly influence the prevalence of childhood malaria in Nigeria [69]. Suitable environmental conditions such as humidity and precipitation create favorable breeding sites for mosquitoes, the vectors responsible for malaria transmission. Temperature also plays a vital role; while higher temperatures can shorten the growth and development of mosquitoes, lower temperatures can increase the risk of malaria transmission. The spatial variability in malaria prevalence across different regions indicates that environmental factors like climate and geography contribute to the distribution of the disease, with higher burdens observed in specific areas.

These factors can interact with each other and the parasite’s genetic diversity to create a complex and dynamic host–parasite relationship, leading to variations in the disease’s severity and outcome. Developing successful malaria prevention and treatment plans requires an understanding of these variables.

## 4. The Role of Oxidative Stress in Malaria Pathogenesis

There are two main ways that Plasmodium infection causes oxidative stress (OS): through the breakdown of hemoglobin and as a result of the host immune response, which causes phagocytes to produce reactive oxygen species (ROS) and reactive nitrogen species (RNS). Systemic and tissue oxidative damage, especially to the brain and lungs, can result from this process. However, OS can be both beneficial and detrimental. While it plays a role in parasite eradication, excessive OS marked by elevated malondialdehyde (MDA) levels can increase disease severity. Research has shown that children with severe malaria have enhanced OS, which is typified by lower hemoglobin concentrations, decreased ascorbate, and raised MDA levels. As previously reported, OS has also been linked to the onset and progression of cerebral malaria [20]. It contributes to the inflammatory response by influencing the development and activity of dendritic cells (DCs) in response to *P. falciparum* infection. OS causes lipid peroxidation on the surface of erythrocytes, which exacerbates the disease caused by malaria [70]. It is difficult to fully understand the mode of action due to its dual roles in parasite removal and molecular damage [71].

Oxidative stress, characterized by an imbalance between reactive oxygen species (ROS) and antioxidant defenses, is a hallmark of malaria infection. During the intraerythrocytic development of Plasmodium parasites, the host’s red blood cell (RBC) undergoes oxidative damage due to the release of ROS from both the parasite and the host’s immune cells. These ROS target cellular lipids, leading to lipid peroxidation, a process that generates reactive aldehydes such as 4-HNE and MDA [70]. Blood oxidative stress markers play a significant role in the pathogenesis of *Plasmodium falciparum* malaria in non-immune children [22]. These markers, such as malondialdehyde (MDA) and 4-hydroxynonenal (4-HNE), are indicative of oxidative damage and are associated with the severity of malaria symptoms, including anemia and immune response modulation [70]. In cerebral malaria, increased lipid peroxidation correlates with the severity of the disease, as evidenced by elevated levels of MDA in the cerebrospinal fluid (CSF) of patients [72].

Advanced oxidative protein products (AOPPs) are also biomarkers of oxidative stress, formed primarily through the oxidation of proteins, particularly albumin, fibrinogen, and lipoproteins. These products are generated under conditions of oxidative stress, where reactive oxygen species (ROS) and chlorinated oxidants, such as those produced by the myeloperoxidase/hydrogen peroxide/halide system, modify proteins. AOPPs are characterized by the presence of dityrosine, carbonyl groups, and cross-linking bonds, which give them unique biological properties similar to advanced glycation end products (AGEs) [73]. They bind to the receptor for AGEs (RAGE), triggering inflammatory and oxidative responses.

AOPPs are present in small quantities under physiological conditions but increase with age and in pathological states, including malaria. Their measurement is a simple and rapid method for assessing oxidative stress and has been proposed as a diagnostic and prognostic tool in various diseases, including malaria [74].

AOPPs have been identified as significant biomarkers of oxidative stress and disease progression in malaria. Studies have shown that plasma AOPP levels are elevated in individuals with Plasmodium falciparum infection and correlate with disease severity, especially in non-immune individuals. It is also associated with clinical manifestations such as anemia and cerebral malaria. The formation of AOPPs in malaria is linked to the breakdown of hemoglobin by the parasite, which generates reactive oxygen species (ROS) through the Fenton reaction. This process creates a highly oxidative environment that damages host proteins and contributes to the pathogenesis of the disease [22].

As a result of oxidative stress, there is a marked reduction in antioxidant markers such as vitamins A and E, glutathione, and enzymatic antioxidants like superoxide dismutase, which leads to an imbalance, creating the need to cushion the effects of OS [75]. One of the proposed strategies for achieving that is through antioxidant supplementation as a therapeutic strategy, which could go a long way in improving outcomes, especially in non-immune children [76]. Another defense line against OS damage is through the anti-oxidative stress pathway genes. These genes encode enzymes and proteins that neutralize ROS and repair oxidative damage.

## 5. Dynamics of Host–Parasite Relationship in Cellular Aging and Malaria

**Figure 1:**
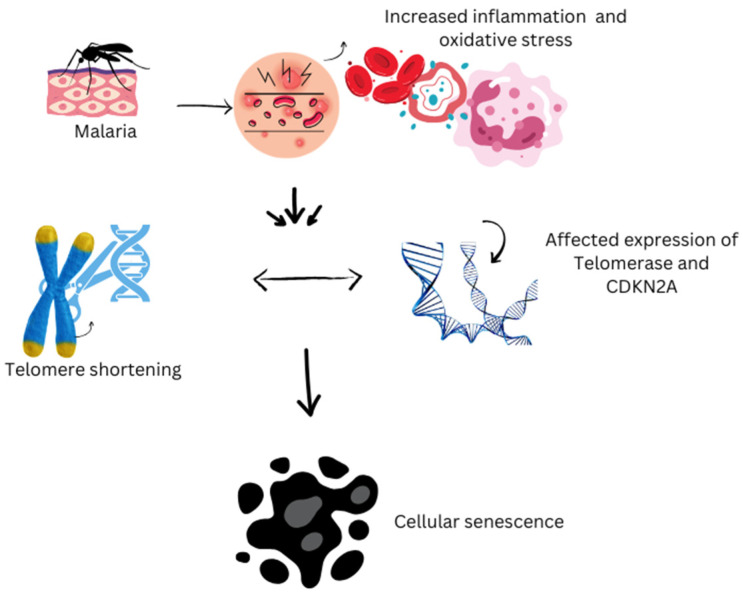
Impact of malaria on cellular aging.

Plasmodium parasites, the causative agents of malaria, can infect humans and other vertebrates, impacting socioeconomic development and causing significant health issues globally. Severe long-delayed malaria caused by *P. malariae* can lead to chronic morbidity and severe complications like severe anemia, cerebral malaria, convulsions, and renal impairment, particularly in older individuals. The host–parasite relationship in malaria can significantly impact aging biomarkers such as telomere length (TL) and the senescence-associated gene CDKN2A expression in diverse blood cells [77,78]. Even at low levels, *Plasmodium falciparum* infection can trigger significant inflammation and oxidative stress, resulting in accelerated telomere shortening and cellular aging (Figure 1). This effect is most evident 1 month post-treatment but is nearly reversed 12 months after infection, provided there is no reinfection [51,77]. Inflammation and oxidative stress are interconnected processes that play key roles in the immune system’s fight against malaria parasites. However, they also contribute to the breakdown of homeostasis and health associated with aging. The controlled human malaria infection (CHMI) model has been used to explore the relationship between telomere length (TL), CDKN2A, inflammation, oxidative stress, and tissue damage before, during, and after low-level exposure to parasites, taking into account individual variability, prior exposure, and co-infections [77,78]. Daily measurements of parasite density and telomere length allowed researchers to pinpoint the exact timing of telomere shortening due to the infection and its recovery following treatment and parasite elimination. The effects of the parasite on aging biomarkers lasted for a month, mirroring findings in travelers with acute *P. falciparum* infections, where the most significant impact occurred one month after treatment. A summary of relevant findings is reported in Table 2.

This figure illustrates the biological impact of malaria on cellular aging processes. Malaria infection increases oxidative stress and triggers inflammatory responses within the body, which disrupt cellular homeostasis. These stressors influence aging biomarkers by accelerating harmful processes such as telomere shortening and the onset of cellular senescence. As telomeres—the protective caps on chromosomes—shorten faster than normal, cells lose their ability to divide and function properly, leading to premature aging at the cellular level. This cascade highlights the critical role of oxidative damage and chronic inflammation in linking malaria infection with the acceleration of aging processes.

### 5.1. Role of Chronic Malaria in Telomere Degradation

Chronic malaria infection in wild birds leads to long-term costs through telomere shortening, affecting survival, lifetime reproductive success, and offspring quality [81]. Individuals with chronic infections experience higher telomere loss rates than uninfected birds, indicating a direct link between malaria infection and telomere degradation. Studies on birds experimentally infected with malaria reveal a positive correlation between the intensity of acute-phase infection and the rate of telomere shortening, emphasizing the link between infection severity and telomere loss [81,82]. The offspring of malaria-infected mothers exhibit shorter telomeres compared to those born to uninfected mothers, indicating that malaria infection may have transgenerational effects on telomere length. Telomere length in offspring appears to be influenced by maternal environmental factors, suggesting that chronic malaria could be a key driver of accelerated telomere attrition and cellular aging in wild bird populations [82]. Telomere degradation paves the way for accelerated cellular aging and senescence. Aside from reduced lifespan and faster telomere shortening in birds, as noted above, it induces high levels of inflammation and oxidative stress, contributing to the degradation of telomeres and cellular senescence [80,82]. As we age, the protective caps at the end of our chromosomes gradually get shorter, but chronic malaria accelerates this process, impacting cellular aging dynamics and reducing lifespan. However, it should be noted that the impact of chronic malaria on telomere degradation is reversible. The protective caps at the end of our chromosomes continuously shorten with age [51].

### 5.2. Implications for Malaria Pathogenesis and Treatment

Accelerated aging processes, such as telomere shortening due to oxidative stress, have been identified as outcomes of malaria infection [78], while the impacts of the host–parasite connection in the setting of malaria are still being studied. This has been hypothesized to decrease immune cell efficiency, especially in susceptible groups like children and the elderly, making it more difficult for them to fight off malaria infection, and may also lead to further complications. A deeper understanding of disease pathophysiology can be gained by researching the host–parasite connection [83,84]. In the context of cellular aging, attempts have been undertaken to identify and comprehend how telomerase may be investigated as a therapeutic target in the treatment of malaria, especially for the creation of innovative treatments [78,85]. Evidence suggests that inhibiting the action of Plasmodium telomerase could result in the death of the parasite, making it a promising target for medication [86]. Moreover, *Plasmodium falciparum* GBP2, a telomere-associated protein, has been demonstrated to interfere with telomere preservation in model systems [87], making it a potentially effective anti-plasmodial drug. Additional proteins that interact with quadruplexes and are linked to telomeres have also been recognized as possible targets for malaria treatment [87].

## 6. Conclusions

Relationships between hosts and parasites are delicate balances in which the parasite gains from the host’s resources while the host may suffer adverse effects like decreased fitness or increased susceptibility to diseases like malaria in the case of the plasmodium parasite. Numerous ecological and health-related processes are fundamentally influenced by this dynamic interaction between the two creatures. To better understand the intricacies of these interactions, researchers need to frequently investigate host–parasite relationships. By holistically examining how parasites take advantage of their hosts and how hosts react to parasitic illnesses, researchers can learn more about immune responses, disease transmission, and evolutionary adaptations in both hosts and parasites, which can provide insights into novel ways to fight malaria. However, all hope is not lost since there are promising new targets that are believed to make a difference in the fight against malaria- telomeres and telomerase alongside their associated proteins, in addition to many others.

## Figures and Tables

**Table 2:** Summary of key studies investigating the effect of malaria on cellular aging biomarkers.

S/N	Study Title	Key Findings	References
1	Biomarkers of cellular aging during a controlled human malaria infection	Investigates the impact of malaria infection on cellular aging markers in humans and discusses the role of telomeres, telomerase, and cellular senescence in the context of malaria infection. These results imply that cellular aging markers, such as telomere length, can be impacted by both acute and chronic malaria infections.	[77]
2	Telomere length dynamics in response to DNA damage in malaria parasites	The process of chromosomal end stabilization and telomere healing in response to DNA damage within the subtelomeric regions is examined in this research, which focuses on telomere length dynamics in malaria parasites. It demonstrates how telomere healing triggers the production of telomere repeats, which ultimately stabilize and resume their usual functions.	[51]
3	Malaria parasites possess a telomere repeat-binding protein that shares ancestry with transcription factor IIIA.	This study explores the telomere-binding protein in malaria parasites, which shares ancestry with transcription factor IIIA (TFIIIA). The research highlights the structural identity of telomeric complexes and their role in maintaining genome integrity.	[79]
4	Cellular aging dynamics after acute malaria infection: A 12 Month Longitudinal Study.	Cellular aging is impacted by acute malaria infection; telomerase activity is decreased and CDKN2A expression is increased. Three months after infection, telomeres were similarly shorter. Following this, there was a decrease in CDKN2A expression, followed by an increase in telomerase activity. Additionally, the reversal of cellular aging was demonstrated by the steady restoration of telomere length over a year.	[80]
5	Biomarkers of cellular aging during a controlled human malaria infection: A 12-month longitudinal study	Age-related telomere shortening, which telomerase can prevent, is associated with increased human diseases. Malaria infections have the potential to accelerate cellular aging, which is associated with age-related disorders and affects telomere length and cellular senescence. Even mild parasite *P. falciparum* infections may impact cellular aging dynamics. Telomeres are essential for maintaining chromosomal integrity. Malaria infection leads to parallel telomere shortening across different body tissues, indicating a systemic impact on cellular aging.	[77,78]
6	Parallel telomere shortening in multiple body tissues owing to malaria infection	Telomere length is correlated with both aging and organismal health; as cells divide, telomeres shorten, signifying the replicative age of the cells. Telomere attrition can result from prolonged stress that inhibits telomerase activity. While control persons did not exhibit any discernible changes over time, experimental individuals infected with malaria had increased telomere shortening in their blood cells. In addition to blood cells, other bodily tissues that experience telomere attrition include the liver, lungs, spleen, heart, kidney, and brain cells.	[81]
7	Chronic infection. Hidden costs of infection: Chronic malaria accelerates telomere degradation and senescence in wild birds	Birds with chronic malaria infections have been found to live shorter lives. Infected birds had a considerably shorter life duration than uninfected birds, which led to a lower lifetime reproductive success rate, according to a study on great reed warblers.	[82]

## Abbreviations

The following abbreviations are used in this manuscript:

ACTs	Artemisinin-based combination therapies
IPTp	Intermittent preventive treatment during pregnancy
SMC	Seasonal malaria chemoprevention
ITNs	Insecticide-treated nets
IRS	Indoor residual spraying
RTS,S/AS01	Mosquirix malaria vaccine
LILRB1	Leukocyte immunoglobulin-like receptor B1
LILRB2	Leukocyte immunoglobulin-like receptor B2
*Pf*EMP1	*Plasmodium falciparum* erythrocyte membrane protein 1
PV	Parasitophorous vacuole
STEVOR	sub-telomeric variable open reading frame proteins
GLURP	Glutamateprich protein
MSPs	Merozoite surface protein
RIFIN	Repetitive Interspersed family proteins
iRBC	Infected red blood cells
miRNA/miR	MicroRNAs
P. ANKA	Plasmodium berghei ANKA strain
ROS	Reactive oxygen species
SMA	Severe Malarial Anemia
HbAS	Sickle cell trait
G6PD	Glucose-6-phosphate dehydrogenase
GWAS	Genome-wide association studies
TL-	Telomere length
CDKN2A	Cyclin-dependent kinase 2A
CHMI	Controlled human malaria infection
DNA	Deoxyribonucleic Acid
TFIIIA	Transcription factor IIIA
CDC	Center for Disease Control
IFN-γ	Interferon-gamma
Th	T-helper cell
HZ	Hemozoin
pRBC	Parasitized red blood cells
DCs	Dendritic cells
NKs	Natural killer cells
MDA	Malondialdehyde
4-HNE	4-hydroxynoneral
AOPP	Advanced oxidative protein products
OS	Oxidative stress
